# Oxidative and Glycation Stress Biomarkers: Advances in Detection Technologies and Point-of-Care Clinical Applications

**DOI:** 10.3390/molecules30214286

**Published:** 2025-11-04

**Authors:** Hiroko Yamaguchi, Hiroshi Yamaguchi

**Affiliations:** 1 Research Institute of Agriculture, Tokai University, 871-12 Sugido, Mashiki, Kamimashiki, Kumamoto 861-2205, Japan; h.kuwata@tokai.ac.jp; 2 Department of Food and Life Science, School of Agriculture, Tokai University, 871-12 Sugido, Mashiki, Kamimashiki, Kumamoto 861-2205, Japan; 3 Graduate School of Agriculture, Tokai University, 871-12 Sugido, Mashiki, Kamimashiki, Kumamoto 861-2205, Japan; 4 Graduate School of Bioscience, Tokai University, 871-12 Sugido, Mashiki, Kamimashiki, Kumamoto 861-2205, Japan

**Keywords:** oxidative stress, glycation stress, biomarkers, AGEs, biosensors, POC testing

## Abstract

Oxidative and glycation stress are interrelated pathological processes that significantly contribute to the development and progression of chronic diseases, including diabetes, chronic kidney disease, cardiovascular disorders, and neurodegenerative conditions. These processes alter biomolecules by generating reactive oxygen species (ROS), reactive nitrogen species (RNS), and advanced glycation end products (AGEs), thereby amplifying cellular dysfunction. Therefore, precise monitoring of these biomarkers is essential for understanding disease mechanisms and for clinical assessments. Conventional methods, such as chromatography, mass spectrometry, and immunoassays, provide high sensitivity and specificity; however, their extensive clinical application is restricted owing to their high cost, labor intensity, and equipment requirements. In contrast, emerging electrochemical and optical biosensor technologies offer advantages in terms of rapidity, portability, and real-time analysis and hold promise for point-of-care (POC) testing and integration into wearable devices. This review systematically summarizes the detection principles and clinical applications of oxidative and glycation stress-related biomarkers and highlights the need for integrated monitoring systems that can simultaneously capture both processes. Advances in these technologies are expected to contribute significantly to early diagnosis, risk stratification, and implementation of personalized medicine.

## 1. Introduction

Oxidative and glycation stress are interrelated pathological processes that play a central role in the pathogenesis of chronic diseases. Oxidative stress is caused by the excessive production of reactive oxygen species (ROS), such as superoxide anion (O_2_^−^), hydrogen peroxide (H_2_O_2_), and hydroxyl radicals (•OH), and reactive nitrogen species (RNS), including nitric oxide (NO) and peroxynitrite (ONOO^−^). These reactive species oxidatively modify lipids, proteins, and nucleic acids, resulting in molecular dysfunction, inflammation, and various types of cell death, including apoptosis and ferroptosis (an iron-dependent form of regulated cell death characterized by excessive lipid peroxidation) [1,2,3].

In contrast, glycation stress originates from the non-enzymatic Maillard reaction, in which reducing sugars such as glucose and fructose react with protein amino groups. The early products, Schiff bases and Amadori intermediates, undergo subsequent oxidation (glycoxidation) and dehydration-condensation reactions, ultimately yielding advanced glycation end products (AGEs) [4,5,6]. AGEs irreversibly alter protein structure and function and activate the receptor for AGEs (RAGE), thereby triggering chronic inflammation, fibrosis, and additional oxidative stress [7,8,9]. Moreover, glycation stress is not limited to the reduction in sugar levels. Glycolytic intermediates, such as glyceraldehyde-3-phosphate (GA3P) and dihydroxyacetone phosphate (DHAP), degrade into highly reactive carbonyl compounds, including methylglyoxal (MGO) and 3-deoxyglucosone (3-DG), which strongly promote AGE formation [10,11].

**Figure 1:**
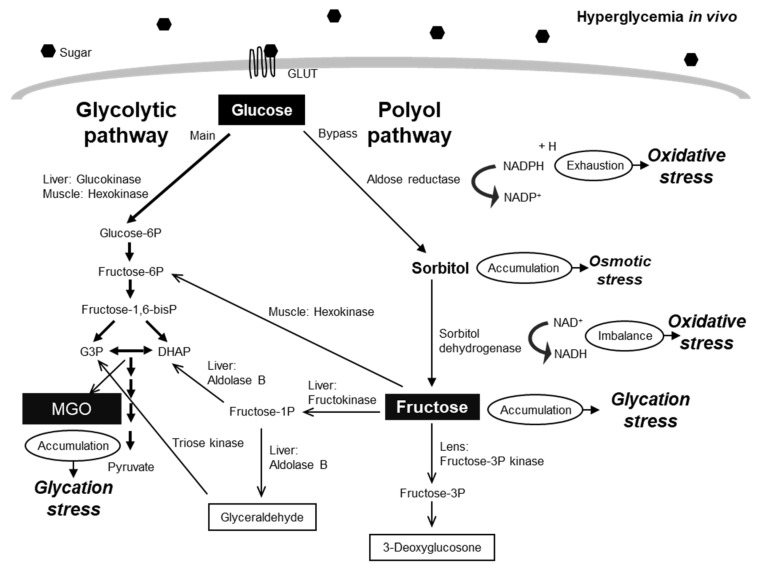
Crosstalk between oxidative and glycation stress in disease progression. Hyperglycemia enhances glucose flux through the polyol pathway, where aldose reductase reduces glucose to sorbitol, which is subsequently oxidized to fructose. Fructose metabolism promotes the formation of reactive dicarbonyl compounds and AGEs, while simultaneously generating oxidative stress via NADPH depletion and ROS production. These processes create a positive feedback loop that amplifies both oxidative and glycation stress, contributing to the onset and progression of diabetic complications.

There is growing evidence that oxidative and glycation stress are pathologically related. Oxidative stress accelerates the conversion of Amadori products into AGEs through glycoxidation, whereas AGEs and reactive carbonyl species disrupt mitochondrial oxidative phosphorylation, leading to increased ROS production [12,13]. Furthermore, AGE–RAGE interactions activate NADPH oxidase, thereby enhancing oxidative stress [14,15]. This oxidative–glycative cycle is now recognized as a key mechanism driving the progression of diabetes, chronic kidney disease, neurodegenerative disorders, and atherosclerosis [16] (Figure 1). Despite the well-established interconnections among these stress pathways, biomarker assessments are often performed independently of one another. Currently, no practical diagnostic system is available for the simultaneous and comprehensive monitoring of oxidative and glycation stress in clinical settings. The development of such integrated technologies would enable dynamic assessment of disease processes and offer new opportunities for improved patient management.

In this review, we focus on the major biomarkers of oxidative stress (ROS, RNS, oxidative byproducts, and oxidized albumin), glucose and related metabolites, and glycation stress markers such as AGEs (Table 1). We summarize their detection methods and clinical or point-of-care (POC) applications, with particular emphasis on biosensor technologies, and discuss future perspectives for comprehensive biomarker monitoring systems.

## 2. Oxidative Stress–Related Biomarkers

Oxidative stress is defined as an imbalance between ROS and RNS generation and the antioxidant defense mechanism that eliminates them. This imbalance plays a central role in the molecular pathogenesis of a wide range of disorders, including chronic inflammation, atherosclerosis, diabetes complications and neurodegenerative diseases [17,18]. Recently, significant progress has been made in the development of visualization probes and detection techniques for ROS, protein and DNA oxidation, and lipid peroxidation, which have greatly improved the ability to monitor oxidative stress–related biomarkers [17,18]. Furthermore, oxidized albumin, particularly the form oxidatively modified at the Cys34 residue, has emerged as a promising marker for kidney disease progression, indicating its potential for quantitative evaluation [19,20,21]. Time-course analyses of Cys34 oxidation following exercise-induced muscle injury have demonstrated its utility as a marker of dynamic oxidative stress [20,22].

Oxidative stress–related biomarkers can be classified into three main categories. First, the direct measurement of reactive species, including O_2_^−^, •OH, H_2_O_2_, NO, and ONOO^−^. However, these molecules are highly unstable and short-lived, necessitating the use of ultrasensitive fluorescence probes, electrochemical approaches, or biosensors employing nanostructured electrodes for their detection [17,18]. The second category involves the examination of oxidative damage products generated by ROS and RNS. For example, a systematic review revealed that the levels of these products were significantly elevated during the active phase of inflammatory bowel disease [23]. The third category focuses on evaluating oxidatively modified proteins, such as oxidized albumin and oxidized LDL. Studies focusing on Cys34-oxidized albumin are representative examples of this category [19,20,21,22].

This review summarizes the underlying mechanisms, clinical significance, and current detection technologies, with an emphasis on recent biosensor-based approaches for oxidative stress–related biomarkers across three categories: (i) reactive species, (ii) oxidative damage products, and (iii) oxidatively modified proteins.

### 2.1. ROS and RNS

ROS and RNS play crucial roles in intracellular and extracellular signaling and maintenance of homeostasis. However, their excessive accumulation induces oxidative damage to lipids, proteins, and DNA, thereby contributing to the pathogenesis of atherosclerosis, diabetic complications, and neurodegenerative disorders [24,25]. Electron spin resonance (EPR) and fluorescent probes are widely used to detect ROS and RNS. However, EPR is expensive and requires specialized operating conditions, and fluorescent probes suffer from limited specificity, cytotoxicity, and inadequate quantification [26,27].

Recent advances in electrochemical biosensing have enabled the sensitive and practical detection of ROS/RNS. For instance, peroxidase-modified electrodes have traditionally been used for H_2_O_2_ measurements [28], and the incorporation of nanomaterials, such as graphene, carbon nanotubes, and gold nanoparticles, has resulted in high sensitivity and low limits of detection (LOD) [29,30]. Notably, non-enzymatic H_2_O_2_ sensors based on MnO_2_ nanosheets or Pt nanoparticles allow the real-time quantification of µM-level hydrogen peroxide in serum [31].

Electrodes modified with NO reductase or hemoglobin have been investigated for NO detection [32,33]. Recently, carbon electrodes adorned with gold nanoparticles have enabled NO detection in artificial tears at nanomolar concentrations [34]. The integration of these electrodes with microelectrodes and lab-on-a-chip platforms further facilitates the real-time monitoring of NO release from cells and extracellular fluids [35]. Moreover, dual-analyte biosensors have been developed for the simultaneous detection of ROS and RNS. For example, graphene oxide–based electrodes capable of detecting both H_2_O_2_ and NO have been utilized in models of inflammation and ischemia–reperfusion injury, demonstrating their utility for monitoring oxidative stress dynamics [29]. These advancements pave the way for spatiotemporal mapping of oxidative stress, which has been difficult to achieve using conventional assays.

### 2.2. Biomarkers of Oxidative Damage

Oxidative stress induces chemical modifications in lipids, proteins, and DNA. Among the markers of lipid peroxidation, malondialdehyde (MDA), 4-hydroxynonenal (4-HNE), and isoprostanes are widely quantified using HPLC and mass spectrometry (LC-MS/MS, GC-MS) to assess the risk of atherosclerosis and diabetic complications [36,37]. DNA oxidation has been extensively studied by measuring the 8-hydroxy-2-deoxyguanosine (8-OHdG) biomarker in urine and serum to evaluate chronic disease and cancer risk [38,39].

Beyond traditional ELISA and immunohistochemical methods, electrochemical and optical biosensors targeting oxidative damage products have gained prominence [40]. MDA has been classically quantified using thiobarbituric acid reactive substances (TBARS), and a surface plasmon resonance (SPR) biosensor using anti-MDA–protein adduct antibodies has recently enabled the high-sensitivity detection of MDA [41]. Graphene-modified electrodes facilitate the electrochemical quantification of 8-OHdG at the nanomolar level in urine [42]. Furthermore, optical sensors incorporating quantum dots or gold nanoparticles as fluorescent probes outperform conventional ELISA in terms of sensitivity and specificity, thus holding promise for minimally invasive biomarker assays [43]. Next-generation biosensing technologies targeting lipid and DNA oxidation products complement chromatographic and immunoassay techniques, enhancing rapid and sensitive oxidative stress evaluation in clinical settings.

### 2.3. Oxidized Albumin

Albumin, the most abundant plasma protein, is highly susceptible to oxidative modifications and serves as a sensitive marker of systemic oxidative stress. The free thiol group of Cys34 at the *N*-terminus is notably reactive and undergoes redox conversion from the reduced to oxidized form. Elevated oxidized albumin levels have been linked to the severity of hypertension, chronic kidney disease, and liver disorders, supporting its use as a clinical biomarker [44,45].

Traditional methods, such as HPLC and mass spectrometry, remain the gold standards but are constrained by cost and turnaround time for clinical application. Therefore, faster and more practical assays have been developed. ELISA systems that use antibodies specific to oxidized albumin enable the quantitative measurement of serum human nonmercaptalbumin (HNA) [46]. Electrochemical sensors with gold nanoparticle–modified electrodes have been used to quantify albumin redox states within minutes and have been validated using serum samples from patients with kidney disease [47]. Overall, oxidized albumin is a rapid systemic response biomarker for oxidative stress. Advances in immunoassay- and nanomaterial-based biosensors have extended their clinical applicability, complementing conventional chromatographic and MS-based methods and paving the way for next-generation diagnostic platforms.

## 3. Glycation Stress-Related Biomarkers

Glycation stress results from a cascade of nonenzymatic reactions in which reducing sugars interact with proteins, lipids, and nucleic acids. This process contributes to the development of various chronic and age-related diseases, including diabetes, atherosclerosis, chronic kidney disease, and Alzheimer’s disease [48]. During this cascade, Schiff bases and Amadori adducts are initially formed, followed by the generation of reactive carbonyl species, ultimately resulting in the formation of irreversible AGEs [49,50].

AGEs not only alter the structure and function of target proteins but also enhance inflammation and oxidative stress through RAGE-mediated signaling pathways, thereby sustaining disease progression [51,52]. Glycation and oxidative stress are closely interrelated through the process of “glycoxidation,” which accelerates the formation of AGEs [12,53]. Conversely, AGEs and reactive carbonyl species can impair mitochondrial function, increase ROS production, and establish a vicious cycle of oxidative stress [54]. Therefore, accurate measurement of glycation stress biomarkers and a deeper understanding of their dynamics are essential for the prediction, diagnosis, and treatment of various diseases.

This section highlights three major categories of biomarkers associated with glycation stress: (i) glucose and glucose metabolites, (ii) glycated proteins, and (iii) AGEs. For each biomarker, the underlying mechanisms of formation, clinical significance, detection methods, and emerging technologies are described, highlighting recent progress in biosensing and mass spectrometry-based approaches [55].

### 3.1. Glucose and Glucose Metabolites

Glucose is the primary energy source and initial substrate for cellular metabolism. Under normal physiological conditions, blood glucose levels are meticulously regulated by endocrine factors such as insulin and glucagon [56,57]. This regulation is impaired in diabetes and insulin resistance, resulting in persistent hyperglycemia. Chronic hyperglycemia accelerates non-enzymatic glycation, driving the formation of early glycated products, such as glycated hemoglobin (HbA1c) and glycated albumin (GA) [48,58].

Enzymatic electrode-based methods, such as glucose oxidase (GOD)-and glucose dehydrogenase (GDH)-based assays, remain the gold standard for blood glucose measurements, supporting both hospital-based diagnostics and self-monitoring of blood glucose (SMBG) [59]. Recently, paper-based microfluidic devices (µPADs) with immobilized enzymes have been developed, providing an affordable, smartphone-compatible colorimetric detection system with an LOD as low as 0.01 mM [60,61,62].

Continuous glucose monitoring (CGM) through real-time glucose measurement in the interstitial fluid has transformed clinical practice by capturing postprandial spikes and nocturnal hypoglycemia [63]. Emerging minimally invasive or noninvasive devices include microneedle-based biosensors and sweat-based wearable sensors [64,65]. For example, a glucose-responsive microneedle patch has demonstrated closed-loop insulin release and normoglycemic restoration in animal models [66].

In addition, reactive carbonyl species derived from glycolytic intermediates, such as MGO and 3-DG, are potent AGE precursors [67]. Although LC-MS/MS has been the standard method for their quantification, innovative electrochemical biosensors enhanced with nanocarbon and metallic nanoparticles enable the sensitive detection of MGO at nanomolar levels in biological samples [68,69,70].

### 3.2. Glycated Proteins

Glycated proteins are formed when reducing sugars bind non-enzymatically to free amino groups (lysine residues and *N*-terminal sites) [71,72]. Early glycation products, such as Schiff bases and Amadori adducts, serve as intermediate biomarkers that reflect the glycemic status of the body.

HbA1c, formed by the glycation of the *N*-terminal valine of the β-chain of hemoglobin A, reflects mean blood glucose levels over a period of 1–2 months and is a well-established diagnostic marker for diabetes [73,74]. However, its accuracy can be influenced by red blood cell lifespan and hematological abnormalities [75]. Glycated albumin (GA), which forms at the lysine residues of serum albumin, indicates glycemic control over a shorter period (2–3 weeks) because of the shorter half-life of albumin [76]. GA is particularly useful as a biomarker in conditions where HbA1c is unreliable (e.g., pregnancy, renal failure, anemia, and hemolytic disease) [77].

Traditional measurement methods for glycated biomarkers include HPLC, immunoassays, enzymatic methods, and capillary electrophoresis [78]. Recent developments in biosensors have aimed to offer faster and simpler assays. For instance, electrochemical sensors employing antibodies or aptamers specific to HbA1c and microfluidic colorimetric or electrochemical sensors for GA have been reported [79,80,81].

### 3.3. AGEs

AGEs are irreversible end products formed from Amadori intermediates through oxidation, dehydration, and crosslinking reactions, and they play a central role in tissue damage and the aging process [82]. They contribute to protein crosslinking, tissue stiffness, and proinflammatory signaling through RAGE activation [83].

More than 40 distinct AGEs have been structurally identified, including pentosidine, *N*^ε^-(carboxymethyl)lysine (CML), and glucosepane. Pentosidine, which forms a fluorescent crosslink between lysine and arginine, can be detected using HPLC [84]. CML is strongly associated with oxidative stress and is typically quantified using ELISA and MS [85]. GL is generated through the non-enzymatic reaction of fructose, the end product of the polyol pathway, with lysine residues, and elevated levels of GL have been associated with diabetes and vascular complications [86,87]. However, unlike pentosidine, neither CML nor GL exhibit fluorescence, and their quantification relies mainly on ELISA or mass-spectrometry.

In Japan, pentosidine is the only AGE included in routine clinical blood examinations. Non-invasive detection using skin autofluorescence (skin AF) has been developed as a rapid bedside measure of AGE accumulation, although it lacks molecular specificity [88,89]. Recently, LC-MS/MS analysis of AGEs in hair samples has been reported as a minimally invasive molecular approach [90].

Recent advances in immunoassays and biosensors have expanded the scope of AGE detection. Antibody-based ELISAs can detect serum CML at the pg/mL level [91], whereas competitive ELISAs for pentosidine provide simpler alternatives to HPLC [92]. Electrochemical immunosensor that utilize carbon nanotubes have achieved nanomolar sensitivity [93]. Innovative platforms, such as surface plasmon resonance (SPR)-based antibody sensors [94] have enhanced detection sensitivity and specificity.

Given that AGE formation is accelerated not only by hyperglycemia but also by oxidative stress and lipid peroxidation, AGEs play a central role in the interplay between glycation and oxidative stress [12]. Thus, AGEs are not only diagnostic and prognostic biomarkers but also potential therapeutic targets.

## 4. Clinical Applications and POC Testing

The assessment of oxidative and glycation stress–related biomarkers has been used to diagnose and predict the risk of various diseases, including diabetes, atherosclerosis, chronic kidney disease, and neurodegenerative disorders [95,96,97]. Conventional reference methods, such as MS, HPLC, and immunoassays, offer high analytical precision; however, their limitations in assay time, cost, and the requirement for specialized equipment restrict their utility in routine clinical practice, home monitoring, and community environments, where prompt results are crucial [98,99].

POC testing has attracted increasing attention in overcoming these limitations. As outlined in Section 2, electrochemical biosensors for oxidative stress markers such as MDA and 8-OHdG, and in Section 3, skin AF devices for AGEs, and immunoassays utilizing anti-AGE antibodies represent prototypical POC candidates [100]. These approaches offer rapidity, simplicity, and the capability to measure with noninvasive or minimal sample volumes, thus demonstrating significant potential for immediate diagnosis and risk stratification at the bedside or in outpatient settings (Table 2). For glucose and HbA1c, POC devices have already been widely implemented worldwide [101], and their success has accelerated research on extending POC principles to oxidative and glycation stress markers. For example, microfluidic–nanomaterial hybrid platforms have been developed that enable simultaneous multiplexed detection of oxidative stress (e.g., H_2_O_2_, NO) and glycation stress (e.g., AGEs), allowing the integrated assessment of multiple biomarkers for more accurate disease characterization [102].

Furthermore, advances in wearable technology have facilitated the development of systems that continuously monitor patients. Noninvasive biosensors that use sweat or saliva have demonstrated the potential to capture dynamic variations in oxidative and glycation stress in real-time [103,104]. These innovations are driving a paradigm shift from “single time-point measurement” to “real-time monitoring of pathophysiological changes.”

Overall, POC testing for oxidative and glycation stress biomarkers is expected to supplement conventional laboratory assays and establish a new diagnostic foundation for preventive medicine, personalized healthcare, and home-based monitoring of patients. Future clinical implementation will depend on addressing the challenges of standardization, quality control, cost reduction, and validation through large-scale multicenter trials and real-world studies [105,106].

## 5. Technical Challenges and Future Perspectives

Despite significant advancements in the measurement of biomarkers related to oxidative and glycation stress in recent years, several challenges persist. The following subsections summarize the major technical limitations and outline the future research directions (Table 3).

### 5.1. Limitations in Sensitivity and Specificity

The extremely short half-lives of ROS and RCS make their direct in vivo detection challenging. Traditional approaches, such as EPR and fluorescent probes, exhibit limited reaction specificity, inaccurate quantification, and a risk of false-positive results [107]. Recent innovations include heme peroxidase-modified electrodes for detecting H_2_O_2_ [108] and graphene-modified nanoelectrodes for real-time NO monitoring [109], which demonstrate enhanced sensitivity and selectivity through enzyme–nanomaterial integration. However, when applied to clinical samples, challenges such as matrix effects and reproducibility of detection limits persist.

### 5.2. Lack of Standardization

Biomarkers such as AGEs and oxidized albumin show considerable variability in their reported values depending on the detection method and target analyte. For example, CML and pentosidine levels measured by ELISA and LC–MS/MS often show substantial differences [110], highlighting the urgent need for international standardization and establishment of reference ranges. Similarly, for oxidized albumin, ensuring compatibility between HPLC-based approaches and novel biosensor technologies remains a major challenge.

### 5.3. Non-Invasiveness and Real-Time Monitoring

Skin AF is increasingly utilized for noninvasive AGE assessment in clinical practice; however, its limitation is that it measures nonspecific fluorophores rather than defined molecular species [88,103]. Wearable biosensors using sweat or saliva have enabled real-time stress monitoring [102]; however, comprehensive validation data from clinical settings are lacking.

### 5.4. Lack of Integrated Assessment

Currently, no clinically implemented system can simultaneously and comprehensively monitor oxidative and glycation stress. Although biosensors capable of detecting individual markers, such as H_2_O_2_, NO, and AGEs, have been developed as described in Section 2 and Section 3, capturing their dynamic interactions necessitates multiplexing capabilities and integrative analytical frameworks.

### 5.5. Future Perspectives

Future progress is likely to be driven by the following strategies. (i) Application of nanomaterials to develop ultrasensitive sensors using graphene, carbon nanotubes, and metallic nanoparticles [111]. (ii) Integration with microfluidics through multiplexed on-chip devices capable of simultaneously detecting oxidative and glycation biomarkers in small-volume samples [102]. (iii) Noninvasive continuous monitoring through the refinement of wearable devices for sweat, tear fluid, and saliva, coupled with rigorous clinical validation [104]. (iv) AI-driven integrative analytics to develop algorithms for the temporal and multiparametric analysis of oxidative and glycation stress markers to advance personalized medicine [112]. If successfully established, these strategies may enable next-generation diagnostic systems capable of real-time and personalized evaluation of oxidative–glycation stress, thereby contributing significantly to prevention and therapeutic decision-making in diabetes, atherosclerotic disease, and aging-related disorders.

## 6. Conclusions

Oxidative and glycation stress influences the onset and progression of diabetes, chronic kidney disease, cardiovascular disorders, and neurodegenerative diseases. This review outlines biomarkers associated with oxidative stress, including ROS/RNS, lipid peroxidation products, oxidized DNA modifications, and oxidized albumin, and their detection methods (Section 2). Glycation stress–related biomarkers, such as HbA1c, Amadori products, and AGEs, and their analytical technologies are also discussed (Section 3). As highlighted, oxidative and glycation stress–derived molecular species are not independent; rather, they interact closely and collectively to contribute to pathological processes in vivo.

Conventional methods, such as LC–MS/MS, HPLC, and ELISA, continue to be the benchmarks because of their high sensitivity and specificity. However, these approaches are constrained by their high costs, lengthy processing durations, and dependence on specialized infrastructure. In contrast, recent advances have driven the development of biosensors suitable for clinical and POC applications, including electrochemical and optical sensors for MDA and 8-OHdG, noninvasive AGE assessment by skin autofluorescence, and nanomaterial-based sensors that enable the rapid detection of oxidized albumin. These innovations suggest that biosensors can complement or replace conventional methods in specific diagnostic settings. Despite these promising advances, several challenges remain for the practical implementation of biosensors in clinical settings. Issues such as long-term stability, reproducibility between batches, and biocompatibility with complex biological matrices still limit their widespread clinical adoption. Addressing these factors through material innovation, standardized calibration procedures, and clinical validation studies will be essential for successful translation to real-world applications.

However, no integrated system currently exists that can simultaneously and comprehensively monitor oxidative and glycation stress. Growing evidence highlights the significance of evaluating their interactions, for example, through AGE–RAGE signaling, which amplifies ROS generation [113], or their roles in ferroptosis pathways [114,115]. Therefore, future diagnostic and therapeutic monitoring strategies should consider oxidative glycation interactions as a unified system.

Advances in nanotechnology, microfluidic devices, and noninvasive wearable sensors combined with AI-driven multidimensional data integration are expected to enable next-generation diagnostic platforms capable of real-time and personalized assessment of oxidative glycation stress [116,117]. For instance, CGM devices such as Dexcom G7 (Dexcom, Inc., San Diego, CA, USA) and Abbott FreeStyle Libre 3 (Abbott Laboratories, Abbott Park, IL, USA), both of which have undergone extensive clinical validation, demonstrate the feasibility of wearable electrochemical sensing platforms in real-world healthcare settings. Similarly, prototype wearable sweat sensors capable of monitoring oxidative stress biomarkers (e.g., uric acid and lactate) have progressed to early-stage clinical evaluation, underscoring the translational potential of such technologies [118,119]. Furthermore, AI-based predictive modeling and multimodal data fusion approaches are emerging as powerful tools for integrating biochemical, physiological, and imaging data to enhance biomarker monitoring accuracy. Deep learning models have been applied to predict oxidative stress and metabolic risk from multi-omics datasets [120], while machine learning algorithms combining sensor-derived and biochemical data have improved detection and classification performance for glycation-related biomarkers [121].

In this review, the term “Point-of-Care (POC)” refers specifically to diagnostic systems that provide rapid, near-patient results, including both clinically approved bedside devices (e.g., CGM systems such as Dexcom G7 and FreeStyle Libre 3) and wearable or portable prototypes currently under clinical evaluation. In contrast, conventional laboratory-based measurements that require centralized facilities and extended processing times are referred to simply as “laboratory-based methods” throughout the text to distinguish them clearly from POC diagnostics.

Such AI-driven and POC-integrated systems are expected to transform early diagnosis, risk stratification, and therapeutic monitoring of oxidative and glycation stress–related diseases, ultimately advancing clinical applications toward more precise and efficient personalized medicine.

## Figures and Tables

**Table 1:** Oxidative and glycation stress–related biomarkers.

Category	Biomarker	Detection Methods	Clinical Relevance
Oxidative Stress	Lipid peroxidation product	TBARS, LC–MS/MS, electrochemical sensors	Atherosclerosis, progression of diabetes
	Oxidized DNA modification	ELISA, LC–MS/MS, electrochemical sensors	Renal disease, cancer risk assessment
	Oxidized albumin	HPLC, biosensors	Indicator of CKD progression
Glycation Stress	HbA1c	HPLC, immunoassays, POC devices	Diagnosis and management of diabetes
	AGEs	ELISA, LC-MS/MS, HPLC, skin AF	Vascular complications, assessment of aging

**Table 2:** Comparison between Conventional Analytical Methods and Emerging Sensor Technologies.

Method	Principle	Target Analytes	Sensitivity/Specificity	Time Required	Cost	Suitability for POC
LC–MS/MS	Molecular identification and quantification via mass spectrometry	MDA, 8-OHdG, CML, Pentosidine	◎	Hours to 1 day	High	×
HPLC	Separation with UV/fluorescence detection	Pentosidine, MGO, 3-DG	○	Hours	Medium	×
ELISA	Antibody-based immunoassay	CML, Pentosidine, HbA1c	○–◎	Hours	Medium	△
Electrochemical Sensors	Enzyme/antibody-modified nanomaterial electrodes	MDA, 8-OHdG, CML, HbA1c	◎	Minutes to tens of minutes	Low–Medium	◎
Optical Sensors	SPR, fluorescence, quantum dot–based probes	CML, Pentosidine, ROS	◎	Minutes to tens of minutes	Medium–High	○
Skin AF	Measurement of intrinsic skin fluorescence	Fluorescent AGEs (e.g., pentosidine)	△	Immediate	Medium	◎

◎ = Excellent; ○ = Good; △ = Moderate; × = Not suitable.

**Table 3:** Technical Challenges and Future Perspectives.

Challenge	Current Limitations	Future Directions
Sensitivity/Specificity	Short-lived ROS/RCS species lead to false positives	Enhancement of nanomaterial/enzyme-modified electrodes and molecular recognition elements
Standardization	Inconsistent LC–MS/MS and ELISA values; lack of reference ranges	Development of international standards and certified reference samples
Non-invasiveness	Skin autofluorescence lacks molecular specificity	Advancement of wearable sweat/saliva/tear sensors and hair analysis
Real-time Capability	Conventional methods provide only single time-point measurements	Adoption of continuous wearable monitoring systems
Integrated Evaluation	Oxidative and glycation markers measured separately	Multiplex detection platforms and AI-based integration
Clinical Implementation	High cost, reproducibility issues, limited multi-center data	Large-scale clinical validation and cost-reduction technologies

## Data Availability

Not applicable.
